# Risk factors for African swine fever incursion in Romanian domestic farms during 2019

**DOI:** 10.1038/s41598-020-66381-3

**Published:** 2020-06-23

**Authors:** A. Boklund, S. Dhollander, T. Chesnoiu Vasile, J. C. Abrahantes, A. Bøtner, A. Gogin, L. C. Gonzalez Villeta, C. Gortázar, S. J. More, A. Papanikolaou, H. Roberts, A. Stegeman, K. Ståhl, H. H. Thulke, A. Viltrop, Y. Van der Stede, S. Mortensen

**Affiliations:** 1 University of Copenhagen, Faculty of Health and Medical Sciences, Section for Animal Welfare and Disease Control, Grønnegårdsvej 8, 1870 Frederiksberg C, Denmark; 2 European Food Safety Authority, Via Carlo Magno 1A, 43126 Parma, Italy; 3 The National Sanitary Veterinary and Food Safety Authority, Bucharest, Piata Free Press no. 1 Body D1, District 1, Post Code 013 701 Bucharest, Romania; 4 University of Copenhagen, Faculty of Health and Medical Sciences, Section for Veterinary Clinical Microbiology, Stigbøjlen 4, 1870 Frederiksberg C, Denmark; 5 Statens Serum Institut, Department of Virus and Microbiological Special Diagnostics, Artillerivej 5, 2300 Copenhagen S, Denmark; 6 Federal Research Center for Virology and Microbiology, 601125 Volginsky, Russia; 7 SaBio research group at IREC (Universidad de Castilla-La Mancha & CSIC), Ronda de Toledo 12, 13003 Ciudad Real, Spain; 8 Centre for Veterinary Epidemiology and Risk Analysis, UCD School of Veterinary Medicine, University College Dublin, Belfield, Dublin, D04 W6F6 Ireland; 9 Department for Environment Food and Rural Affairs (DEFRA), Exotic Disease Control team, Area 2D, Nobel House, 17 Smith Square, London, SW1P 3JR England; 10 Utrecht University, Faculty of Veterinary Medicine, Yalelaan 7, Utrecht, The Netherlands; 11 National Veterinary Institute, 751 89, Uppsala, Sweden; 12 Helmholtz Centre for Environmental Research GmbH – UFZ, Department of Ecological Modelling, PG EcoEpi, Permoserstr. 15, Leipzig, Germany; 13 Estonian University of Life Sciences, Institute of Veterinary Medicine and Animal Sciences, Kreutzwaldi 62, Tartu, 51006 Estonia; 14 Danish Veterinary and Food Administration, Stationsparken 31–33, 2600 Glostrup, Denmark

**Keywords:** Disease model, Population dynamics

## Abstract

African swine fever (ASF) entered Georgia in 2007 and the EU in 2014. In the EU, the virus primarily spread in wild boar (*Sus scrofa*) in the period from 2014–2018. However, from the summer 2018, numerous domestic pig farms in Romania were affected by ASF. In contrast to the existing knowledge on ASF transmission routes, the understanding of risk factors and the importance of different transmission routes is still limited. In the period from May to September 2019, 655 Romanian pig farms were included in a matched case-control study investigating possible risk factors for ASF incursion in commercial and backyard pig farms. The results showed that close proximity to outbreaks in domestic farms was a risk factor in commercial as well as backyard farms. Furthermore, in backyard farms, herd size, wild boar abundance around the farm, number of domestic outbreaks within 2 km around farms, short distance to wild boar cases and visits of professionals working on farms were statistically significant risk factors. Additionally, growing crops around the farm, which could potentially attract wild boar, and feeding forage from ASF affected areas to the pigs were risk factors for ASF incursion in backyard farms.

## Introduction

In 2007, African swine fever (ASF) spread from the African continent, where the disease is endemic, into Georgia then on through Eastern Europe, reaching the European Union in 2014. During the first years of the epidemic in the EU (2014-early 2017), the disease mainly affected wild boar, with sporadic spill-over to domestic pigs^1^. In 2017, ASF spread to Romania, initially resulting in a small number of outbreaks in domestic pig farms in the county of Satu Mare, which neighbours Hungary and Ukraine. In July 2018, ASF occurred in two counties neighbouring Satu Mare, but also in five counties around the Danube delta close to the Black Sea in the South East part of Romania. In July 2018, 334 outbreaks were detected, mostly in domestic farms, predominantly in the South East. From then on, ASF spread widely in Romania with outbreaks in more than 1,000 domestic pig farms in 2018 and about 2,500 in 2019 (Animal Disease Notification System of the European Commission (ADNS)).

By December 2019, 21 countries had notified outbreaks with the ASFV Genotype 2 that was first introduced in Europe in 2007, through immediate notifications to the World Animal Health (OIE) Information System (WAHIS), including 11 countries in Europe and 10 in Asia, and causing devastating losses to domestic pig production, and affecting wild boar populations.

Several risk factors for ASF incursion has been described; free range pig management, presence of infected farms in the neighborhood, visits by veterinarians or para-veterinarians, and in backyard farms also including swill feeding together with suspected cases of underreporting and “emergency sales”^2^. Furthermore, Olesen *et al*. described potential routes for indirect transmission of ASFV as uncooked pig meat, processed pig-derived products, feed, matrices contaminated with ASFV and blood-feeding invertebrates^3^. However, often these assessments were based on relatively small numbers of outbreak investigations, due to the small numbers of affected pig farms in each country. For instance, based on retrospective analysis of 26 outbreaks in domestic pig farms in Estonia, Nurmoja *et al*.^4^ found larger farms to be at higher risk of getting infected with ASF and concluded that there was a tendency that multiplier and farrow-to-finisher farms had higher risk of infection. In contrast, based on data from the Russian Federation in the period 2007 to 2012 and using spatio-temporal modelling techniques, small scale farms were found to be at higher risk of infection with ASFV^5^.

Based on the work of Olsevskis *et al*.^6^, the most likely routes of transmission to domestic farms were swill feeding, contact with wild boar and indirect transmission by people having contact with infected farms or use of fresh grass or crops. This study was based on retrospective investigation of 32 outbreaks in domestic farms in the country at that time. In Russia, analysis of all ASF outbreaks until mid-2012 highlighted the importance of swill feeding for the introduction of infection to new pig populations (primary outbreaks). In contrast, the source of spread to secondary cases were often unknown. In those cases where the source of infection was clear, each of the following was implicated: contaminated vehicles, direct contact with pigs or people from pig farms, or introduction of pigs in the period close to outbreak detection^1^.

In addition, several experimental studies have shown that ASF virus can survive long periods in pork, especially in chilled or frozen products where ASF virus has been found after >100 days^7^. Furthermore, mechanical transmission of ASF virus by stable flies over short distances has been described^8,9^. Moreover, several experimental studies have shown that infection of pigs with ASF virus is possible by providing feed spiked with ASF virus^10,11^. The epidemiological relevance of the feeding and vector experiments remains to be clarified.

Terminology throughout the paper will follow usual way of recording. Wild boar diagnosed with ASF are designated *cases*, and domestic pigs included in farm-level *outbreaks*^12^. Domestic operations were classified according to the Romanian herd register i.e. backyard farms registered as “backyards”, and commercial farms either “type A commercial” or “commercial”. In the Romanian herd register, backyard farms were defined as small farms with low levels of biosecurity and production for own consumption only, type A farms were defined as medium sized farms with some biosecurity procedures and these farms can deliver animals to commercial abattoirs, and commercial farms were farms with high level of biosecurity and these farms can also deliver animals to commercial abattoirs.

The purpose of the current study was to investigate factors that could significantly contribute to the risk of occurrence of ASF to domestic backyard or commercial pig farms in Romania. The large number of outbreaks allowed us to use a matched case-control design. Results of this study may help prevent the transmission of ASF in domestic pigs.

## Results

**Figure 1:**
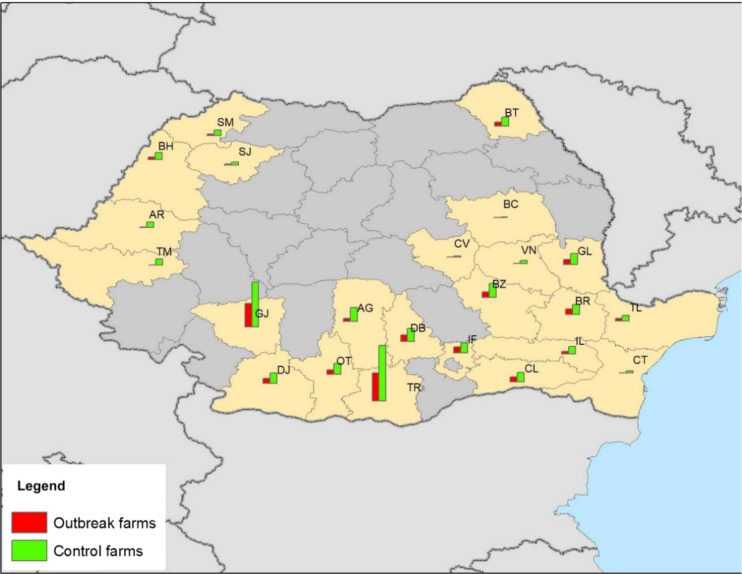
Map of Romania with counties included in the case-control study in yellow. The size of the bar reflects the numbers of case or control farms in each county. (Map created in ArcGIS 10.4.1 for Desktop https://desktop.arcgis.com/en/arcmap/10.4/get-started/installation-guide/existing-arcgis-desktop-users.htm).

In total, 655 farms from 23 counties were included in the analyses: 200 outbreak farms (9 type A, 14 commercial farms and 177 backyard farms) and 455 control farms (32 type A, 70 commercial farms and 353 backyard farms). Control farms were located in all 23 counties within the study area, whereas some counties with outbreaks in the past (BC, CT, CV, TM and VN) did not have any outbreaks during the study period i.e. outbreaks diagnosed between May 15, 2019 and September 15, 2019. The number of outbreak farms in each ASF affected county varied from 1 to 49, with a median of eight. County Giurgiu (GR) and Teleorman (TR) had 41 and 49 outbreak farms included, respectively. In all other counties, the number of included outbreak farms was 11 or less (Fig. 1).

Interviews were performed as soon as possible after ASF was detected on the outbreak farm to minimize recall bias. In total, 580 farms (88.5%) were visited within 10 days after ASF confirmation (for control farms after ASF confirmation in the matching outbreak farm), 41 farms (6.3%) were visited between 11–21 days and 34 farms (5.2%) after more than 21 days with a maximum of 78 days.

### Backyard farms: Descriptive Statistics

#### Observations related to the wild boar-domestic pig interface in the backyard farms

Of the 530 backyard farms, 34 farmers had observed wild boar around the farm. Four farmers had observed crossbred pigs, e.g. striped piglets as a result of a wild boar mating a domestic sow. Three farmers had observed wild boar carcasses around the farm, and three farmers reported that wild boar had access to feed storage. On 21 farms, wild boar had access to the bedding storage, and 114 farms had crops around the farm that could be attractive to wild boar (Table 1).

**Table 1:** Descriptive statistics and univariate analyses for potential risk factors in backyard farms in Romania.

		Case	Control	OR^a^	C.I.	p
**Measures of wild boar around the farm**						
Wild boar seen	Yes	21	13	5.64	2.24–14.3	0.0003
	No	156	340			
Crossbred pigs	Yes	4	0			
	No	173	353			
Wild boar carcass seen	Yes	3	0			
	No	174	353			
Wild boar access to feed storage	Yes	3	0			
	No	174	353			
Wild boar access to bedding storage	Yes	15	6	6.59	2.17–20.0	0.0009
	No	162	347			
Attractive crops	Yes	48	66	2.39	1.31–4.39	0.0047
	No	129	287			
**Sources of drinking water**						
Tap water	Yes	25	60	0.67	0.34–1.31	0.24
	No	152	293			
Fountain water	Yes	25	41	0.76	0.41–1.4	0.38
	No	152	312			
Tank water	Yes	2	0			
	No	175	353			
Surface water	Yes	4	0			
	No	173	353			
**Source of feed and bedding**						
Pigs introduced in HRP	Yes	4	1	8	0.89–71.6	0.063
	No	173	352			
Swill feeding	Yes	28	37	3.89	1.48–10.3	0.0059
	No	149	316			
Cereals	From ASF +	40	25	5.68	1.95–16.6	0.0015
	From ASF-	115	293	0.56	0.28–1.13	0.108
	No	22	35			
Forage	From ASF +	47	23	9.94	4.04–24.5	5.69e-07
	From ASF-	46	142	0.61	0.32–1.15	0.124
	No	84	188			
Farm milling and mixture	From ASF +	25	12	12.49	2.84–54.8	0.0008
	From ASF-	47	151	0.44	0.26–0.73	0.0034
	No	105	190			
Bedding	Straw	47	137	0.33	0.19–0.59	0.0002
	No straw*	130	216			
**Ticks and biting midges observations by farmer**						
Ticks	Yes	9	8	4	1.03–15.6	0.0459
	No	168	345			
Biting midges	<10	40	53	2.27	0.98–5.28	0.0569
	10–100	81	204	0.67	0.41–1.09	0.1108
	>100	56	96			

*wood chips, “others”, none.

^a^OR indicates the odds ratio estimated by a logistic regression matched on the case to which the controls were selected, but with one explanatory variable. Not provided for variables, where 0 observations were found for one of the groups.

#### Observations related to the source of drinking water in the backyard farms

Four farms used surface water (water from a river, pond or lake) and 2 farms used tank water (water stored in a reservoir on the holding) as drinking water for the pigs, while most farmers used tap water or fountain water (pumped up from ground water) (Table 1).

#### Observations related to the source of pig feed and bedding in the backyard farms

In total, 65 farmers fed swill to the pigs, and 5 farmers (four outbreak farms and one control farm) had introduced new pigs to the farm in the high-risk period, i.e. two weeks prior to ASF diagnosis on farm. Cereals, forage, and ingredients for farm milling and mixture were often from areas with ASF (Table 1).

#### Observations during the high-risk period in the backyard farms, e.g. the two weeks before ASF was diagnosed

The total number of professionals visiting the backyard farms during the high-risk period, such as consultants, private veterinarians, maintenance workers, in outbreak farms, ranged from 0 to 10 in outbreak farms with a median of 0 and a 75^th^ percentile of 1, while this range was 0 to 4 in control farms with a median and a 75^th^ percentile of 0 (Supplementary, Fig. S3). The numbers of vehicles entering the farm area ranged from 0 to 40 with a median of 0 and a 75^th^ percentile of 2, while the numbers of non-professional visitors ranged from 0 to 100 with a median of 1 and a 75^th^ percentile of 3. As there was no statistical difference between outbreak farms and control farms, the numbers of vehicles entering and the non-professional visitors are presented for all backyard farms.

#### Observations on co-variates related to the size and location of backyard farms

**Figure 2:**
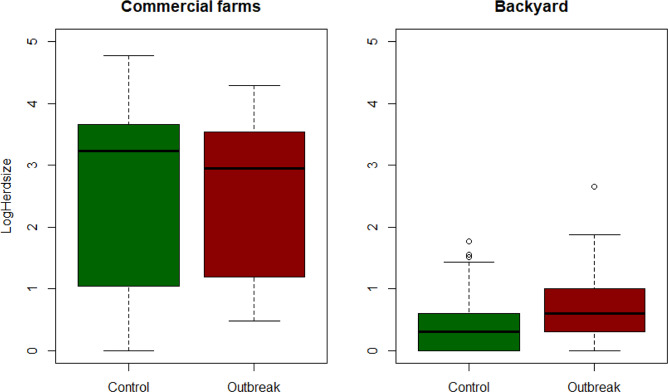
Log(HerdSize) by disease status in Romanian farms.

Outbreak farms were significantly larger than control farms, with a median of 4, a 75^th^ percentile of 10, and 454 as a maximum, while control farms had a median of 2, a 75th percentile of 4 and a maximum of 59 (Fig. 2).

The abundance of wild boar around outbreak farms compared to control farms, i.e. the median wild boar densities, were 0.011 and 0, respectively, while the 75^th^ percentiles were 0.227 and 0.085, respectively. The median farm densities were 2.64 and 3.96 around outbreak and control farms, respectively, while the 75^th^ percentiles were 4.76 and 5.78, respectively. Generally, forest coverage was limited, i.e. <7% forest around 90% of all the farms. All backyard farms included in the study had water bodies (inland and marine waters) within 1 km.

**Figure 3:**
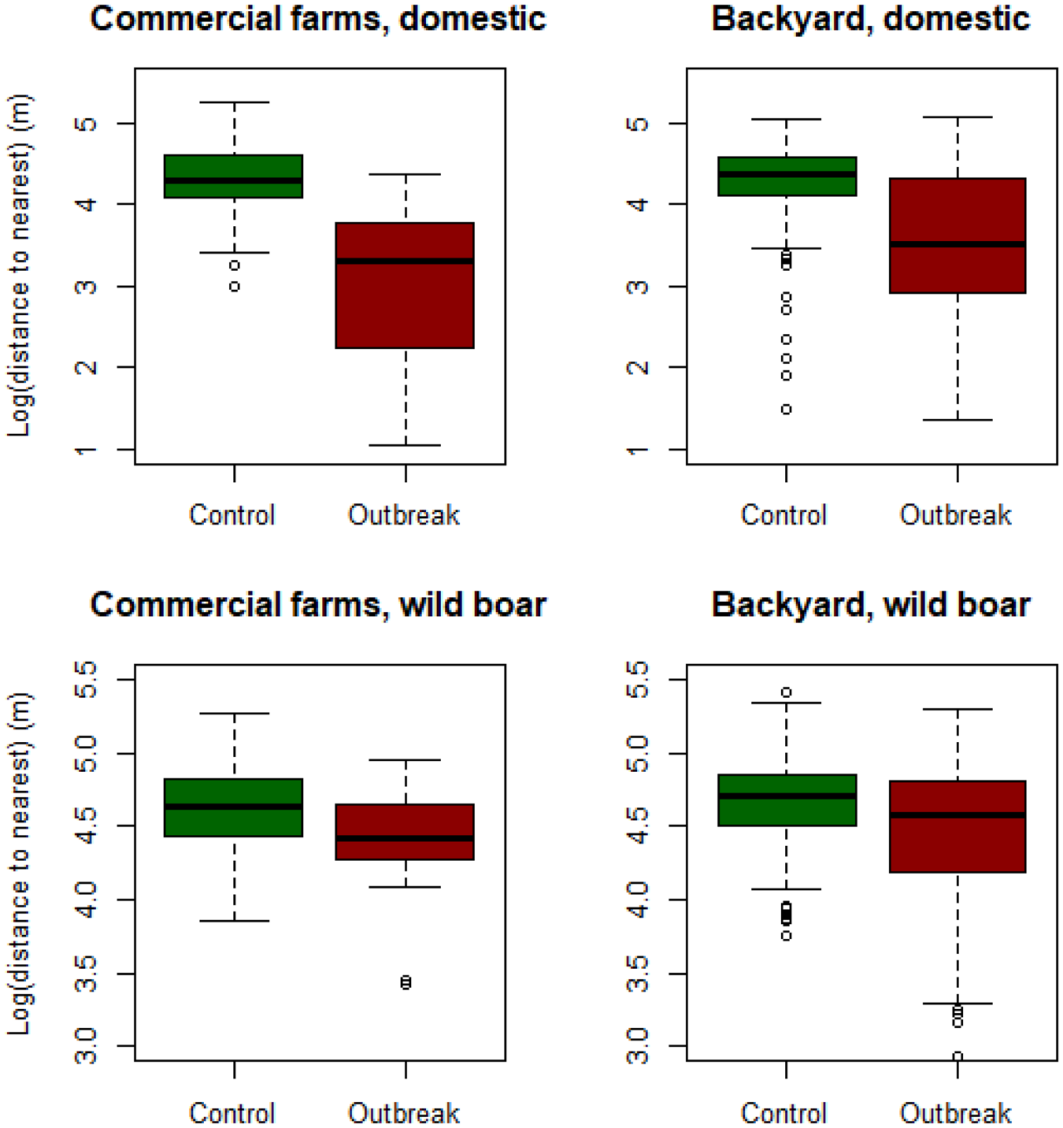
Logarithm of the distance (m) from commercial and backyard farms included in the Romanian case control study to nearest wild boar case or to nearest outbreak in domestic pigs.

Moreover, the median distances to wild boar cases in the high-risk period were 38 km and 51 km for outbreak and control farms, respectively, while median distances to outbreaks in domestic farms in the high-risk period were 3.3 and 24 km, respectively (Fig. 3). The median numbers of outbreaks in domestic farms in the high-risk period within a distance of 2 km were 0 for outbreak farms and for control farms, while the 75^th^ percentiles were 2 and 0, respectively, and the maximums were 9 for outbreak farms and 3 for control farms (Supplementary, Fig. S2).

### Backyard farms, Multivariable Analysis

In the final logistic regression model (Table 2), the logarithm of the number of pigs on the farm, the numbers of outbreaks in domestic pig farms within 2 km, wild boar abundance and the number of professional visitors on the farm remained as significant risk factors, whereas the logarithm of the distance to the nearest outbreak in domestic pigs and wild boar case were protective factors, i.e. the risk of ASF occurrence decreased with increasing distance to the nearest outbreak/case. Furthermore, if attractive crops were grown around the farm and if forage from areas affected by ASF was used on the farm, the risk of ASF occurrence was higher, while the use of straw as bedding material had a protective effect.

**Table 2:** Final model. Risk factors for ASF occurrence in Romanian backyard farms.

		Unadjusted		Adjusted			
		OR	c.l.	OR	c.l.	p	
Ln(HerdSize)		5.23	3.17–8.66	28.18	7.21–110.2	1.58e-06	***
WB abundance		2.49	1.49–4.15	5.036	1.36–18.6	0.0153	*
Ln(nearWB)		0.31	0.21–0.44	0.222	0.079–0.629	0.00459	**
Ln(nearDB)		0.419	0.34–0.52	0.613	0.379–0.992	0.0464	*
DP2		12.49	5.09–30.7	4.601	1.34–15.8	0.0155	*
Professional visits in HRP		2.44	1.73–3.43	6.93	3.08–15.6	2.8e-06	***
Attractive crops	Yes	2.39	1.31–4.39	9.092	1.85–44.8	0.00665	**
	No	1	—	1	—	—	
Origin of forage	ASF +	9.95	4.04–24.47	19.1	3.52–103.7	0.00063	***
	ASF-	0.61	0.32–1.15	0.627	0.195–2.02	0.435	
	No forage	1	—	1	—	—	
Bedding	Straw	0.33	0.19–0.59	0.135	0.0378–0.485	0.00212	**
	NoStraw	1	—	1	—	—	

**Figure 4:**
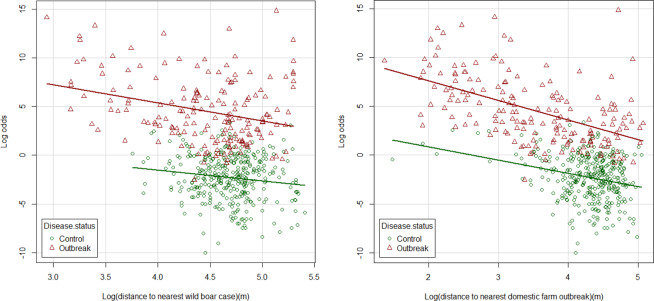
Model prediction of occurrence of ASF in backyard farms as function of the logarithm to the nearest case of ASF in wild boar (left) or the logarithm to the nearest outbreak of ASF in domestic farms (right), by disease status.

Figure 4 shows the predicted logarithm of the odds for farms included in the study as a function of the distance of the farm to the nearest wild boar case or outbreak in a domestic farm. The plots show that distances to wild boar cases and outbreaks in domestic farms in general are longer in control farms compared with outbreak farms. This can be seen by most of the control farms (green dots) located on the right side of the two plots.

### Commercial farms, Descriptive Statistics

As described in the introduction, commercial farms are here defined as farms registered as “commercial” or “type A commercial” in the Romanian herd register.

#### Observations related to wild boar-domestic pig interface in the commercial farms

On commercial farms, crossbred piglets and carcasses of wild boar were never seen on or around the farm, and wild boar did not have access to feed or bedding storage. However, some commercial farmers did observe wild boar in the area around the farm, and attractive crops were often grown around the farm (Table 3).

**Table 3:** Descriptive statistics and univariate analyses for potential risk factors in commercial farms in Romania.

		Case	Control	OR	C.I.	p
**Measures of wild boar around the farm**						
Wild boar seen	Yes	3	6	2.68	0.57–12.6	0.21
	No	20	96	1	—	—
Attractive crops	Yes	8	55	0.43	0.15–1.23	0.12
	No	15	47	1	—	—
**Sources of drinking water**						
Tap water	Yes	2	8	0.93	0.14–5.98	0.94
	No	21	94	1	—	—
Fountain water	Yes	19	87	0.81	0.23–2.89	0.75
	No	4	15	1	—	—
Tank water	Yes	3	7	2.37	0.59–9.62	0.23
	No	20	95	1	—	—
Surface water	No	23	103	—	—	—
Tap water	Yes	2	8	0.93	0.14–5.98	0.94
**Biosecurity measures**						
Pigs introduced in HRP	Yes	4	29	0.51	0.15–1.74	0.29
	No	19	73	1	—	—
Swill feeding	Yes	2	4	5.48	0.3–99.4	0.25
	No	21	98	1	—	
Fencing	Yes	23	101	—	—	—
	No	0	1			
Not entering premise at carcass collection*	Yes	3	18			
	No	7	18			
Non-contact loading of animals*	Yes	2	4			
	No	8	32			
Locker room*	Yes	1	7			
	No	9	29			
Rodent control*	Yes	3	7			
	No	7	29			
Intact insect nets*	Yes	1	4			
	No	9	32			
**Ticks and biting midges observations by farmer**						
Ticks	Yes	0	1			
	No	23	101			
Biting midges	<100	16	78	0.398	0.11–1.41	0.15
	>100	7	24			

*Answered by all 41 Type A farms and 5 commercial farms.

#### Observations related to the source of drinking water in the commercial farms

None of the commercial farms used surface water, 10 used tank (stored) water, 10 used tap water (mains water) and 106 farms used fountain water (ground water) as drinking water for the pigs (Table 3).

#### Observations related to the source of pig feed and bedding in the commercial farms

Six commercial farms used swill feeding, which included two outbreak farms. Only two farms (both outbreak farms) used cereals or ingredients, for on farm milling and mixture, from ASF-infected areas (Table 3). In total, 34 commercial farms used bedding, of which three were outbreak farms. Straw was used in 12 farms, of which 2 were outbreak farms.

#### Observations during the high-risk period in the commercial farms, i.e. 4 weeks (type A farms) or 6 weeks (commercial farms) before ASF was diagnosed

The total number of professionals visiting the commercial farms in the high-risk period, such as consultants, private veterinarians, maintenance workers, ranged from 0 to 90, with a median of 1. The numbers of vehicles entering the farm area ranged from 0 to 96, with a median of 2, while the numbers of non-professional visitors ranged from 0 to 20 with a median and 75^th^ percentile of 0. Eighty percent of the commercial farms did not have non-professional visitors in the high-risk period.

About one quarter of the farms had introduced pigs to the farm in the high-risk period, and no commercial farms had used boars from other farms in the high-risk period.

#### Observations related to biosecurity

Nearly all commercial farms were fenced (Table 3). The questions on biosecurity were only answered by 46 farms; all of the 41 “type A” farms included in the study and five commercial farms. Of those answering the biosecurity questions, approximately half of them had no persons entering the farm when carcasses were collected for rendering, while only six farms used non-contact loading of animals, 8 farms used locker rooms, and five farms had intact insect nets on windows (Table 3).

#### Observations on co-variates related to the size and location of commercial farms

Commercial farms ranged from 24 to 60,066 pigs, with a median of 3,107, while type A farms ranged from 1 to 106 pigs, with a median of 6. The distribution of herd size for the combination of commercial and type A farms is shown in Fig. 1.

Wild boar abundance around commercial farms ranged from 0 to 1.51 wild boar per km^2^ with a median of 0.046, while farm density ranged from 0 to 10.3 farms per km^2^, with a median of 2.18. Generally, forest coverage was limited around all the farms, i.e. <5.9% forest around 85% of the farms. Only two commercial farms included in the study did not have water bodies within 1 km.

The median distances to wild boar cases was 27 and 39 km from outbreak and control farms, respectively, while the median distances to outbreaks in domestic farms were 2 and 20 km, respectively (Fig. 3). Three control farms had one outbreak farm within 2 km, while 9 outbreak farms had 1–2 outbreaks within 2 km, and 2 outbreak farms had, within 2 km, 3 and 10 outbreaks, respectively.

### Commercial farms, Multivariable analysis

In the final logistic regression model (Table 4), the distance to the nearest outbreak in domestic pigs was the only significant risk factor, with an OR of 0.182 (0.071–0.45). In other words, there is a decreasing risk of ASF occurrence with increasing distance to the nearest outbreak in a domestic pig farm. The logarithm of the distance between commercial farms and the nearest ASF outbreak is shown as a boxplot in Fig. 3 (upper left) for outbreak and control farms, respectively.

**Table 4:** Final model. Risk factors for ASF occurrence in Romanian commercial farms.

	OR	c.l.	p	
Ln(nearDP)	0.182	0.0711–0.465	0.000369	***

## Discussion

From 2007, when ASF entered Georgia, until the first case was detected in EU in 2014, the disease was mainly seen in domestic pig farms with low levels of biosecurity^13^. Since 2014, Chenais *et al*.^13^ describe direct transmission between wild boar as well as indirect transmission between wild boar via the habitat, suggesting that the wild boar-habitat cycle has dominated ASF spread in eastern EU, with occasional spillover to domestic farms. This contrasts with the experience in Romania since summer 2018, where many outbreaks in domestic farms, primarily with low levels of biosecurity, have been diagnosed, while relatively few cases in wild boar were notified^14^. Therefore, this study included covariates targeted to estimate and statistically evaluate the exposure from wild boar sources and from outbreaks in other domestic farms on study farms.

According to Romanian legislation, farms are classified as backyard, type A or commercial farms with different biosecurity requirements. Therefore, for practical and logistical reasons, two analyses were performed, one for backyard farms and one for type A and commercial farms. Furthermore, different types of questions were used in accordance with the type of farm, e.g. questions on biosecurity would most often not be relevant for backyard farms. However, as herd types can be difficult to compare between countries, with each country defining herd types differently, in order to extrapolate and to compare between countries or areas, classification of herd types based on numbers of pigs would often be of more use.

In the study period, too few commercial farms became infected to reach the estimated sample size, and therefore, even with the extra control farms included, the power of the study was only strong enough to determine a single statistically significant risk factor.

In backyard farms, nine explanatory variables remained in the final multivariable model. Of these, five factors were related to the numbers of and distances to potential sources of ASF virus in the area, i.e. number of outbreaks in domestic pig farms within 2 km, the distance to the nearest outbreak in a domestic pig farm, the distance to the nearest case of ASF in wild boar, wild boar abundance in the area surrounding the farm, and the growing of crops near the backyard which are attractive to wild boar. Three factors were related to farm management, i.e. the number of professional visitors in the high-risk period, the use of straw as bedding and the use of forage from ASF + areas. And finally, the larger size of the backyard farm itself was a risk factor. In commercial farms, only one factor remained in the final model, i.e. the distance to the nearest outbreak in another pig farm.

### Factors related to exposure from other outbreak farms and cases in wild boar

The proximity of both outbreaks in domestic farms and/or wild boar cases were shown to be significant risk factors for ASF incursion in both Romanian backyard and commercial farms. The influence of outbreaks in domestic pigs in the model reflected the larger number of outbreaks in Romania compared to other European countries. However, the proximity of outbreaks in domestic farms and wild boar cases should be considered a proxy for the presence of ASF virus in the area. In other countries, with more wild boar cases and fewer outbreaks in domestic farms, the relative contribution of wild boar cases and wild boar abundance compared to outbreaks in domestic farms would most likely be more important. However, both covariates together are considered a proxy of the presence of ASF virus in the areas, and therefore we expect the results to be of value also for these countries.

In this study, ASF virus circulation in domestic farms in the proximity to the study farm was presumed to be correlated with the number of outbreaks in domestic pig farms within 2 km, the distance to the nearest outbreak in a domestic pig farm, and the density of pigs and farms. Similarly, the risk of ASF virus from wild boar surrounding the farm was presumed to be correlated with the distance to the nearest case of ASF in wild boar, wild boar abundance in the area surrounding the farm, wild boar seen in the surroundings of the farm, and the growing of crops attractive to wild boar near the backyard. In the final multivariate model, ASF virus in domestic farms were described by the number of outbreaks in domestic pig farms within 2 km, the distance to the nearest outbreak in a domestic pig farm. The risk of ASFV from wild boar surrounding the farm was described by the distance to the nearest case of ASF in wild boar, wild boar abundance in the area surrounding the farm, and the growing of crops attractive to wild boar near the backyard. The sensitivity of the surveillance system in domestic pig farms is expected to be high, as clinical surveillance must be performed in all pig farms in the zones around outbreak farms on a regular basis, while the sensitivity of the surveillance in wild boar is expected to be lower. It has been stated that “nothing is easier than to ignore a rotten, smelly wild boar carcass in a forest”^15^, and that the goal in passive surveillance of wild boar should be to find and investigate 10% of the carcasses^15^. This difference in surveillance sensitivity between domestic farms and wild boars contributed to decision-making during model building. Because the sensitivity of wild boar surveillance is lower, we sought to include several indirect measures of wild boar presence in the model, rather than focusing solely on direct measures, such as detected wild boar cases. Martinez-Lopez *et al*.^16^ found that communes with a number of closed herds above the median on Sardinia to have increased risk of ASF. However, their analysis was performed on commune level and therefore distance to other outbreaks was not included in the model.

For the calculation of the nearest distance to outbreaks in domestic pigs and wild boar cases, only outbreaks and cases diagnosed in the high-risk period were included. This was based on the assumption that outbreaks and cases diagnosed after the farm was infected could not have influenced the risk of ASF incursion. The high-risk period of six weeks in commercial farms was based on mortality data from one large commercial farm (unpublished data). However, the time from infection to detection in Estonian outbreak farms was estimated to be short, i.e. <7 days^4^, while Guinat *et al*. described by simulation studies that ASF virus could be circulating for nearly a month before mortality increased markedly^17^, and Halasa *et al*. similarly simulated the time between infection and detection to a median of 24 days^18^. Furthermore, in the analyses of Estonian data, all wild boar cases within a year before a farm was diagnosed with ASF were included in the analysis. The exact duration of the high-risk period can be difficult to determine, due to the short clinical phase and high mortality of ASF^19^, which especially in large farms can lead to an increase in herd mortality which is not immediately recognized as a consequence of ASF. If the length of the high-risk period is overestimated, this can lead to inclusion of risk factors, which in reality could not have influenced disease incursion. In contrast, an underestimation of the length of the high-risk period could potentially result in important risk factors being missed. Still, even with this uncertainty, the distance to the nearest domestic outbreaks and wild boar cases as well as the number of outbreaks in the area around the farm can be interpreted as a proxy for the amount of ASF virus circulating in the area.

### Factors related to human activity and management

In this study, the number of professional visitors during the high-risk period was identified as a risk factor for ASF occurrence in backyard farms (Table 1). Similar results were found in a matched case-control study from Nigeria^20^. And experimental studies have indicated that indirect transmission of ASF virus to pigs introduced to a contaminated pen can only happen shortly after the pen has become contaminated (after 1 days)^21^. This suggests that swine professionals visiting several backyard farms on the same day may have contributed to the incursion of ASF.

The feeding of fresh forage (e.g. hay or grass) to pigs in backyard farms that had been harvested in areas affected by ASF was found to be a significant risk factor (Table 1). This is an important outcome and similar observations have been reported from Latvia and Lithuania^2^. In combination with growing attractive crops around the farm, this could explain the seasonal character of the disease. Seasonality of ASF in domestic pigs has been a clear observation of the ASF epidemic in the EU^14^.

In contrast, the use of straw was negatively correlated with the incursion of ASF in backyard farms in the model. The reason for this is unclear. It could be hypothesized that this was a confounding factor related to other husbandry factors, which could have protected the farms for ASF incursion but were not included in the interview. In addition, it could be hypothesized that the probability that straw contains infectious ASF virus is very low, due to the longer harvesting period, in generally warm weather conditions, which could inactivate the virus, as compared to freshly cut grass. ASF virus has previously been shown to be transmitted to pigs eating stable flies (*Stomoxys calcitrans*) spiked with ASF virus^8^. It could be hypothesized that use of straw as bedding would attract fewer flies than concrete floors with manure, or that straw could lead to activation of the pigs, and following that the probability of the pigs catching and eating flies or finding dead flies on the ground would decrease. However, this is very speculative and needs more investigation.

ASF virus can survive for long periods in meat from infected pigs^7^, and swill feeding has frequently been implicated in the spread of ASF^7,22^, especially as the source of initial introduction into new countries^23^. Furthermore, a study on the relation between hypothesized risk factors and ASF on commune level in Sardinia showed that a high number of closed farms per commune was related to higher risk of ASF, and the authors suggested that swill feeding might be more common in closed small-scale farms^16^. Although swill-feeding was statistically significant in the univariate analyses, it did not remain in the final multivariate model. This contradiction could be based on the feeding of swill that did not contain pork or at least did not contain ASF virus. However, there has been a total ban on swill-feeding in EU since 2002^24^; no animal protein can be fed to the same species (Article 22 of Regulation 1774/2002/EC), e.g. no pig protein or catering waste (i.e. waste food originating in restaurants, catering facilities and kitchens, including central kitchens and household kitchens) can be fed to pigs. Still, in this study we found that 71 Romanian farms fed swill to pigs, especially backyard farms. Therefore, further initiatives to explaining the regulation and the risk related to swill feeding is needed.

### Herd size as a risk factor

Among Romanian backyard farms, outbreak farms were significantly larger than control farms. A survival analysis from Estonia also showed that large farms were at higher ASF risk^4^. However, the Estonian farms categorized as large were 101–1000 pigs or >1000 pigs, while in our study, herd size was included as continuous variable and backyard and commercial farms were analyzed separately. In our study, herd size in outbreak farms originated from ADNS data, while herd size in control farms originated from the questionnaires. These different sources are not expected to cause differences, as the numbers in ADNS from outbreak farms were reported shortly before or at the same time as the questionnaires were filled for control farms. Furthermore, the relation between ASF occurrence and herd size was in accordance with the experience from the Romanian official veterinarians carrying out the epidemiological outbreak investigations in outbreak farms.

Herd sizes overlapped between backyard farms and commercial farms, with up to 454 pigs in the largest backyard farm and no more than 24 pigs in the smallest commercial farm. Furthermore, type A farms, which in the analyses were categorized as commercial farms, were somewhat in between in size, ranging from 1 to 106 pigs. Our differentiation between backyards and commercial was based on current herd types in the Romanian herd register and on which level of biosecurity could be expected in each type of farm. Furthermore, we took into account the relevance of different types of questions for each type of farm, e.g. questions on biosecurity would most often not be relevant for backyard farms. However, in order to extrapolate and to compare to other countries or areas, classification of herd types based on numbers of pigs would often be clearer. On the contrary, small-scale farming varies in different areas of Europe, from the free-ranging extensive production system of Sardinia^25^ to pot-bellied pigs kept as pets in Northern Europe. Additionally, the numbers of commercial outbreak farms were limited, leading us to increase the numbers of controls per outbreak to have some statistical power in the analyses of the questionnaires completed on commercial farms. Still, it was only possible to show statistical significance of one variable, i.e. the logarithm of the distance to the nearest outbreak in domestic pigs. This finding might be caused by the limited numbers of commercial outbreak farms included, i.e. the statistical power of the analyses is too small to reveal other risk factors.

### Possible transmission routes involved in ASF incursions in romania

Although the final model included several risk factors describing the virus load in the surroundings, we still lack knowledge on how the virus is transmitted from the surroundings into the farm. Many of the transmission routes, which could bring the virus from the surroundings into the farm, were already included in the study, but were not statistically significant (Supplementary Table S1). However, other transmission routes, such as the farmer carrying the virus in boots or equipment, were not included for backyard farms. Furthermore, aerosol transmission from pen to pen has been demonstrated in experimental studies^10^, and the common stable fly (*Stomoxis calcitrans*) has been shown to transmit ASF virus mechanically by biting susceptible pigs or by being eaten by pigs^8,9^.

It was not possible to perform an entomological survey on the outbreak and control farms. Therefore, during each interview, estimates were made of the number of ticks in cracks on the wall and in the ground of the pig sheds as well as on the pigs, and the numbers of biting midges and mosquitoes observed on the farm, as a proxy of the number of vectors on the farm.

Neither biting midges nor mosquitoes were significant in the analyses. However, flying insects have previously been shown able to act as mechanical vectors over short distances^8,9^, DNA from ASF virus have been found in insects at ASF infected farms^26^, and the seasonality in domestic farms might indicate importance of insects^14^. Therefore, further field studies on the role of insects in ASF spread are needed.

Although the number of ticks (both on the animals and in the environment) was relatively higher in the outbreak farms (Tables 1 and 2) and borderline significant in the univariate analyses, this was not a significant risk factor in the multivariate analyses. Soft ticks are most often nest parasites, meaning that they reside in sheltered environments such as burrows, caves or cracks. Therefore, soft ticks are not expected to influence the risk of ASF introduction into pig farms. As hard ticks do not replicate ASF virus^27^, ticks of this type can theoretically be expected only to be able to act as mechanical vectors^27,28^.

## Conclusion

The results of this study showed that close proximity to outbreaks in domestic farms was a risk factor in commercial as well as backyard farms in Romania. Furthermore, in backyard farms, the number of domestic outbreaks within 2 km around farms, wild boar abundance around the farm, short distance to wild boar cases and growing crops around the farm, which could potentially attract wild boar were statistically significant risk factors, and these risk factors could be interpreted as a proxy for the circulation of ASF virus in the surroundings. Additionally, herd size, visits of professionals working on farms and feeding forage from ASF-affected areas to the pigs were risk factors for ASF incursion in Romanian backyard farms.

## Materials and methods

### Study design

For this study, a matched case-control design was chosen. From May 15, 2019, to September 15, 2019, all domestic pig farms diagnosed with ASF were included as case (outbreak) farms in the study if located in the study area and if no direct link with another outbreak farm had been registered (e.g. as a result of animals being moved from a previously affected outbreak farm).

The study area was defined as Romanian counties with ASF occurrence in wild boar or domestic pig holdings prior to the study period. This included the following counties: Braila (BR), Constanta (CT), Calarasi (CL), Bihor (BH), Buzau (BZ), Vrancea (VN), Tulcea (TL), Galati (GL), Arad (AR), Timis (TM) Satu Mare (SM), Salaj (SJ), Teleorman (TR), Covasna (CV), Ialomita (IL), Giurgiu (GR), Botosani (BT), Bacau (BC), Dambovita (DB), Olt (OT), Arges (AG), Ilfov (IF) and Dolj (DJ) (Fig. 1).

Outbreak farms were matched to control farms. All type A and commercial farms diagnosed with ASF in a specific week and located in the study area were included as outbreak farms. For each type A outbreak farm, 5 type A controls were randomly selected using a random number algorithm, matched on county. Due to the limited numbers of commercial farms in each county, for each commercial outbreak farm we randomly selected 5 commercial controls without matching on county. For backyard farms, a maximum of 9 outbreak farms per county per week were randomly selected, and for each backyard outbreak farm, 2 controls were randomly selected in the same county. Each control was selected and visited in the week following the confirmation of outbreak in the matched outbreak farm. The required sample size for each herd type was estimated to 468 farms, based on an odds ratio of 2.5, a prevalence of exposure among control farms of 0.1, a power of 0.9 and a ratio of outbreak to control farm of 2. As this goal was reached already on August 14^th^, 2019, the inclusion of backyard farms was stopped at this point. However, as few type A and commercial farms were included at that time, the study continued to the end of the study period for these two herd types. Control farms were farms not diagnosed with ASF during the period of the study.

### Data collection

In total, 665 farms were included in the case-control study. Nine control farms, including three backyard farms and six commercial farms, were diagnosed with ASF less than 28 days after being interviewed as controls. These farms were therefore interviewed (again) as outbreak farms, and excluded as controls, as we could not exclude the possibility that the farms might have already been infected at the time of the first interview. Furthermore, one farm did not have pigs present at the day of the visit and was therefore excluded from the analyses.

All farms included in the study were contacted by an official veterinarian (OV) and invited to participate in the study. The OVs visited all study farms and filled out a questionnaire regarding potential risk factors related to management and biosecurity measures implemented on the farm. The questionnaire consisted of 42 questions with the following themes: number and age groups of animals (6 questions), practice of slaughter, outdoor access and other species (5 questions), whether there were wild boar around the farm area (6 questions), feed and water (8 questions), indirect contacts, i. e. vehicles and visitors (3 questions), bedding, manure and fencing (4 questions), contacts to other farms in the high-risk period (6 questions) and observations of ticks, mosquitoes and midges (4 questions). The full questionnaire is available in the Supplementary Materials. Each interview lasted approximately 30 minutes for backyard and 60–90 minutes for commercial holdings. Some questions were related to the high-risk period, interpreted as the time period in which ASF introduction presumably occurred. From outbreaks in other countries, very short time periods between ASF introduction and detection have been estimated, e.g. 2–4 weeks in Latvia^6^, 5–14 days in Russia in 2008–2012^29^, and 1 week from first clinical sign to diagnosis in Estonia^4^. For backyard farms, the high-risk period was therefore defined as 2 weeks, while for commercial farms, this period was defined as 6 weeks, based on mortality data from a very large commercial farm (data not published). Given their intermediate herd size, the high-risk period for type A farms was defined as 4 weeks. For outbreak farms this period, was calculated as 2, 4 or 6 weeks prior to the date of initial ASF detection. For control farms, the same time period as in the matching outbreak farm was used, i.e. starting from the detection date of the outbreak farm and counting backwards for 2, 4 or 6 weeks.

Based on data from the EU Animal Disease Notification System (ADNS), geographic coordinates of all outbreaks in domestic pigs and cases of ASF in wild boar in Romania and neighbouring countries were extracted. For both outbreak and control farms, the distances to other outbreaks during the high-risk period were calculated. With these calculated distances, some covariates were defined to be used in the final logistic regression model namely i) the logarithm of the distance to the nearest wild boar (WB) case, ii) the logarithm of the distance to the nearest outbreak in domestic pigs (DP) and iii) the total numbers of cases in wild boar or outbreaks in domestic pigs, respectively, within 1, 2, 5 and 10 km. Additionally, data on the density of domestic pigs, based on data provided by the National Sanitary Veterinary and Food Safety Authority of Romania, were calculated. The number of pigs per hunting ground and the relative wild boar abundance were calculated based on the numbers of hunted wild boar divided by the size (in km_2_) of the hunting ground in the hunting season 2018–19. Hunting data were provided by the Romanian Ministry of Waters and Forests to calculate the relative wild boar abundance as the number of hunted wild boar per hunting ground. The percentage of forest cover in each hunting ground unit, in which outbreak and control farms were located, was calculated. The presence of water bodies within 1 km of the outbreak or control farms was determined as a binomial variable (yes/no). The percentage of forest cover and presence of water bodies were each extracted from the 2018 raster version of CORINE Land Cover data.

### Data analysis

All data were analyzed using conditional logistic regression models with disease status (outbreak or control farm) as the outcome and the co-variates described above as explanatory variables. In these models, control farms were matched to outbreak farms by herd type and county (not commercial farms). Two models were built, one for backyard farms and one for commercial farms and type A farms. Commercial farms and type A farms will from here on be named “commercial farms”.

First, we described all explanatory variables using descriptive statistics. Where possible, depending on the number of observations, we tested each of the variables in the model, one by one, as univariate analyses.

Secondly, we included all possible explanatory variables in the multivariate models. Stepwise backward elimination was used manually, combined with forward selection, meaning that the predictive variable with the highest p-value was excluded from the model, one by one. For each round, we re-introduced the last excluded predictive variable, in order to test whether it was statistically significant without the last excluded variable. Furthermore, when the final model was found by this procedure, all excluded predictive variables were added one by one, to see if any were now statistically significant and affected the size of the effect of the others. Meaningful interactions were tested, i.e. interactions between the numbers of pigs, whether wild boars were seen around farms, wild boar abundance and pig density and all parameters in the final model. Furthermore, pairwise interactions of the predictive variables in the final model were tested.

The model was run in R, version 3.5.2 “Eggshell Igloo” (R Core Team, 2018), using the survival (logistic regression) and the geosphere (distance calculations) packages. The concordance describes the agreement between an observed response and a predictor. The concordance for the multivariate logistic regression model on backyard farms was 0.929, se = 0.021, while the concordance for model on commercial farms was 0.931, se = 0.04. The R^2^ was 0.429 (max possible = 0.519) for the model on backyard farms and 0.323 (max possible = 0.444) for the model on commercial farms.

### Ethics approval and consent to participate

No animal experiment were included in this study, and therefore approval by institutional review boards was not required. However, the study protocol was approved by the National Sanitary Veterinary and Food Safety (ANSVSA) in Romania together with a European Food Safety Authority (EFSA). Farmers selected for the study were first informed about the questionnaire and asked for their permission to enter the holding and for their willingness to participate. It was made clear to all participating farmers that they could withdraw from the study at any time point, and that all information would be anonymized in all outputs from the study. The farmers were informed that the National Sanitary Veterinary and Food Safety (ANSVSA) in Romania together with a European Food Safety Authority (EFSA) was performing a study to find out more about African swine fever, and informed consent was obtain from all participants. No person under the age of 18 years was interviewed and appropriate guidelines and regulations were followed throughout the study.

Yves Van der Stede, Sofie Dhollander, José Cortinas Abrahantes and Laura C. González Villeta are currently employed with the European Food Safety Authority (EFSA) in the ALPHA Unit that provides scientific and administrative support to EFSA’s scientific activities in the area of Animal Health and Welfare. The author Alexandra Papanikolaou is employed with the European Food Safety Authority (EFSA) in the Unit Evidence Management that provides scientific and administrative support to EFSA’s scientific activities in the areas of data management and analysis. However, the positions and opinions presented in this article are those of the authors alone and are not intended to represent the views or scientific works of EFSA. To know about the views or scientific outputs of EFSA, please consult its website under http://www.efsa.europa.eu.

## Supplementary information

Supplementary information.

Questionnaire
