# Supplementary material for: Risk factors for African swine fever incursion in Romanian domestic farms during 2019
Source: Sci Rep. 2020 Jun 23;10:10215. doi: 10.1038/s41598-020-66381-3 (PMC7311386; doi:10.1038/s41598-020-66381-3)
Supplement: Supplementary file 1 — Supplementary information. [file 41598_2020_66381_MOESM1_ESM.pdf]

Supplementary Information

Risk factors for African swine fever incursion in Romanian domestic farms during 2019

Boklund, A.[1], Dhollander, S.[2], Chesnhoiu Vasile, T.[3], Abrahantes, J.C.[2], Bøtner, A.[4a&4b], Gogin, A.[5], Gonzalez Villeta, L.C.[2], Gortázar, C.[6], More, S.J.[7], Papanikolaou, A.[2], Roberts, H.[8], Stegeman, A.[9], Ståhl, K.[10], Thulke, H.H.[11], Viltrop, A.[12], Van der Stede[2], Y., & Mortensen, S[13].

Supplementary, Table S1: Covariates included in the analyses of risk factors for ASF incursion in Romanian farms, backyard s as well as commercial farms.

Covariate	Type	Description
Total.pigs	Numeric, discrete	Total numbers of pigs on the farm. For case farms, data from ADNS was used
Piglets	Numeric, discrete	Total numbers of piglets on the farm. For case farms, data from ADNS was used
Sows	Numeric, discrete	Total numbers of sows on the farm. For Case farms, data from ADNS was used
Seen.WB	Dichotom, y/n	Does the farm owner see sign of wild boar around the farm
Crossbred.pigs[1]*	Dichotom, y/n	Does the farmer observe crossbred piglets, as result of a wild boar mating a domestic sow
WB Carcass[1]*	Dichotom, y/n	Does the farmer observe wild boar carcasses around the farm
WB.access.to.feed.storage[1]*	DicWB hotom, y/n	Does wild boar have access to the feed storage
WB.access.to.bedding.storage*	Dichotom, y/n	Does wild boar have access to the bedding storage
Attractive.crops	Dichotom, y/n	Are there crops around the farm, which would be attractive to wild boar (maize, fruit trees, oak trees)
Origin.of.the.forrage*	Categorical, no/from ASF-area/from non-ASF area	From where do the forage used for pigs originate
Origin.of.the.cereals*	Categorical, no/from ASF-area/from non-ASF area	From where do the cereals used for pigs originate
Origin.of.the.on.farm.milling.and.mixture*	Categorical, no/from ASF-area/from non-ASF area	From where do the ingredients for home mixing for pigs originate
Vehicles.visits.HRP	Numeric, discrete	Numbers of vehicles entering the farm area in the high risk period (HRP)
Professionals.visits.HRP	Numeric, discrete	Numbers of professional visits in HRP
Nonprof.visitors.HRP	Numeric, discrete	Numbers of non-professional visits in HRP
Bedding	Categorical, straw/no straw	Originally, the type of bedding was registered as no/straw/wood chips/other
Fenced.holding*	Dichotom, y/n	
Manure.from.other.holdings*	Dichotom, y/n	Are manure from other holdings used on the fields around the farm
Pigs.introduced.in.HRP	Dichotom, y/n	Were pigs introduced in HRP
Soft.ticks*	Categorical, 0/0-4/5-9	Are soft ticks observed on the farm
Hard.ticks[1]*	Categorical, 0/0-4/5-9	Are hard ticks observed on the pigs
Ticks	Dichotom, y/n	Combined from the two above
Mosquitoes	Dichotom, y/n	Combined from 0/0-9/10-10/>100
Biting.midges	Dichotom, y/n	Combined from 0/0-9/10-10/>100
Fatteners	Numeric, discrete	Total numbers of fatteners on the farm. For Case farms, data from ADNS was used
Bovine	Dichotom, y/n	Cattle on the farm
Ovine	Dichotom, y/n	Sheep on the farm
Caprine*	Dichotom, y/n	Goats on the farm
Poultry	Dichotom, y/n	Poultry on the farm
Equine*	Dichotom, y/n	Horses on the farm
Pets	Dichotom, y/n	Pets on the farm
Swill	Dichotom, y/n	Are there sign of swill feeding at the farm visit
Compound	Dichotom, y/n	Is compound feed used
Fountain	Dichotom, y/n	Drinking water supply for pigs fully or partly from fountain
Tap	Dichotom, y/n	Drinking water supply for pigs fully or partly from the tap
Tank[2]	Dichotom, y/n	Drinking water supply for pigs fully or partly from a tank
Surface[2]*	Dichotom, y/n	Drinking water supply for pigs fully or partly from surface water

[1] Strongly correlated to other covariates, and were therefore left out of the model

[2] No control farms used surface water and the covariate was therefore left out of the model

Water[2]	Dichotom, y/n	This one takes the lowest level from above assuming tap>fountain>tank>surface (as several farms had >1 type of supply)
**Covariates calculated based on register data from other sources (not answered in the questionnairs)**		
**Covariate**	**Type**	**Description**
nearWB	Numeric, continous	Distance (m) to nearest outbreak in wild boar in the HRP
lnnearWB	Numeric, continous	Ln distance (m) to nearest outbreak in wild boar in the HRP
WB1	Numeric, discrete	Number of wild boar outbreaks within a distance of 1 km from the farm
WB2	Numeric, discrete	Number of wild boar outbreaks within a distance of 2 km from the farm
WB5	Numeric, discrete	Number of wild boar outbreaks within a distance of 5 km from the farm
WB10	Numeric, discrete	Number of wild boar outbreaks within a distance of 10 km from the farm
nearDP	Numeric, continous	Distance (m) to nearest outbreak in domestic pigs in the HRP
lnnearDP	Numeric, continous	Ln distance (m) to nearest outbreak in domestic pigs in the HRP
DP1	Numeric, discrete	Number of domestic pig outbreaks within a distance of 1 km from the farm in the HRP
DP2	Numeric, discrete	Number of domestic pig outbreaks within a distance of 2 km from the farm in the HRP
DP5	Numeric, discrete	Number of domestic pig outbreaks within a distance of 5 km from the farm in the HRP
DP10	Numeric, discrete	Number of domestic pig outbreaks within a distance of 10 km from the farm in the HRP
WBabundance	Numeric, continous	Wild boar abundance around the farm (wb per km^2)
Farmdens	Numeric, continous	Farm density around the farm (farms per km^2)
Pigdens	Numeric, continous	Pig density around the farm (pigs per km^2)
Forest coverage	Numeric, continous	Percentage of forest in the hunting ground where the farm is located
Water in 1 km	Dichotom, y/n	Water bodies (river, sea, lake) within a distance of 1 km from the farm location

*Marks covariates, which could not be tested for commercial farms, as there were none or too few commercial farms with this management to include it in the model.

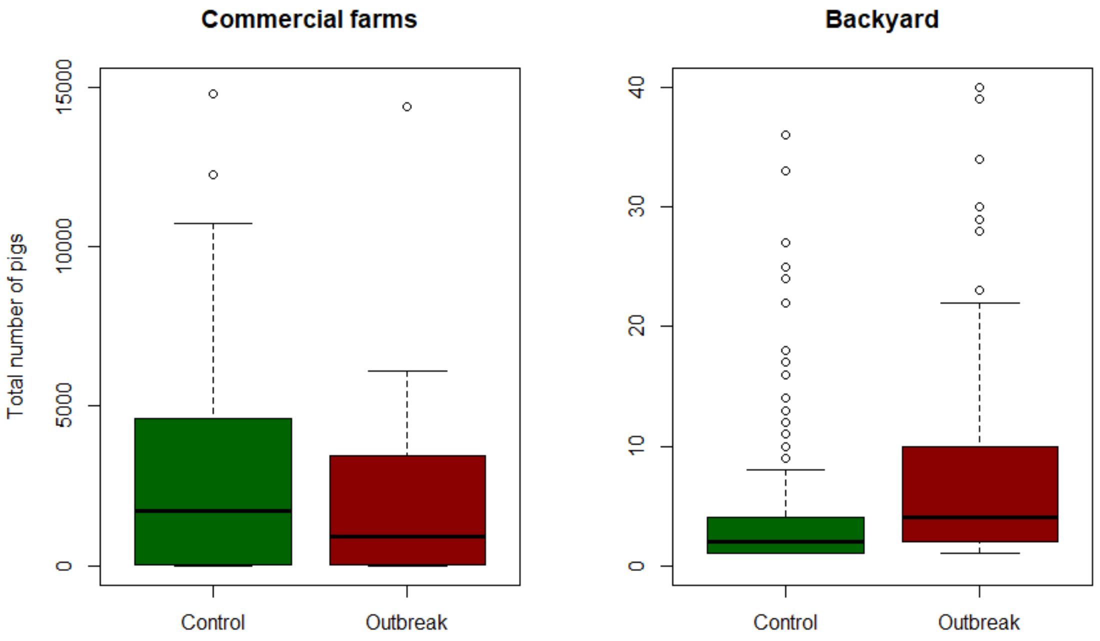

Supplementary, Figure S1: Total number of pigs in Romanian outbreak and control farms in a matched case-control study (for backyards values >40 not shown – 8 observations, 7 cases, 1 control, for commercials values >15,000 not shown – 11 observations, 10 controls, 1 case)

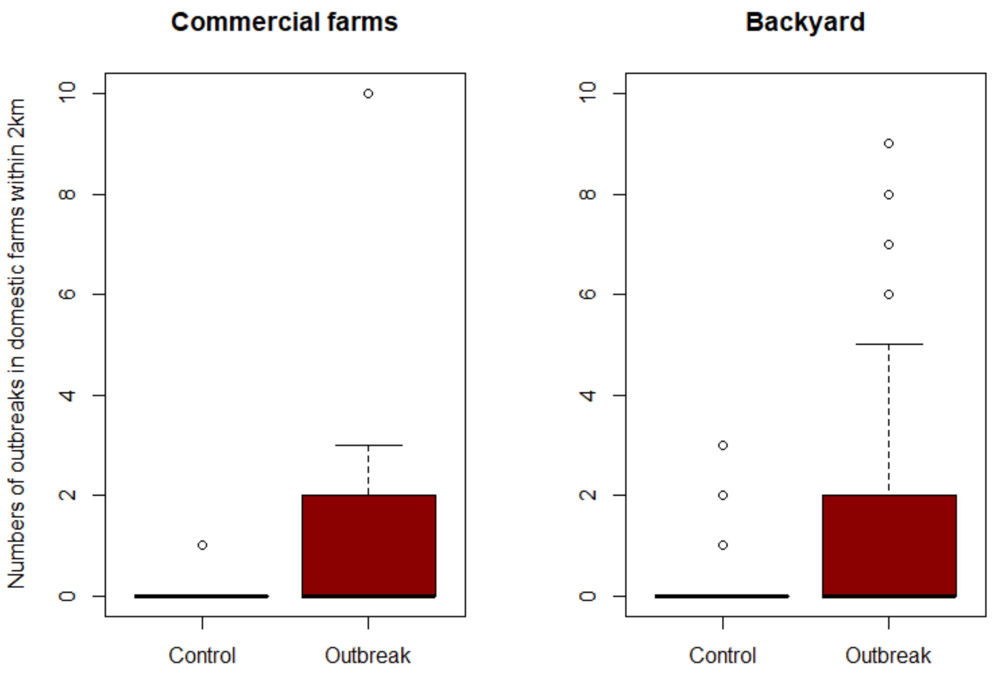

Supplementary, Figure S2: Numbers of outbreaks in domestic farms in the high-risk period within 2 km from Romanian outbreak and control farms included in a matched case-control study.

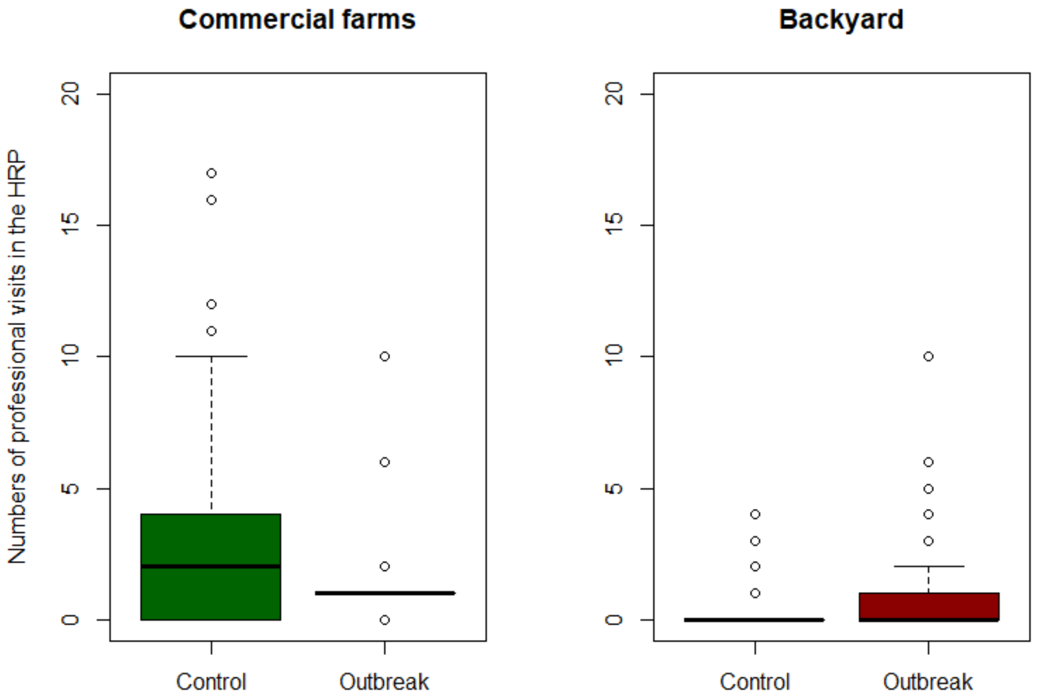

Supplementary, Figure S3: Numbers of professional visits in the high-risk period in Romanian outbreak and control farms included in a matched case-control study

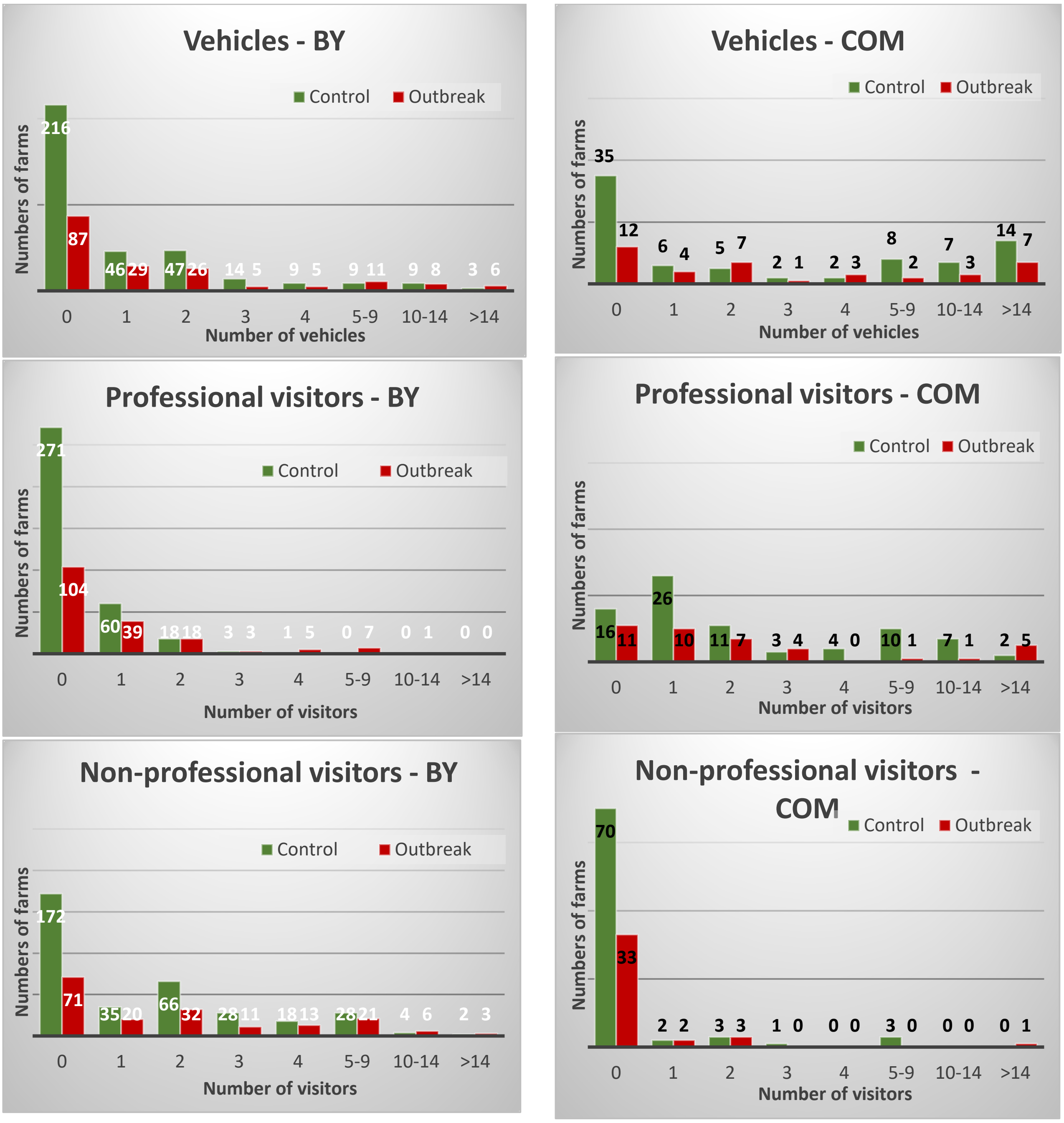

Supplementary, Figure S4: Distribution of the numbers of vehicles, professional visitors and non-professional visitors entering the Romanian farms in the high-risk period, backyard farms left, commercial farms right, y-axis display the number of farms.
