# Supplementary material for: Risk factors for African swine fever incursion in Romanian domestic farms during 2019
Source: Sci Rep. 2020 Jun 23;10:10215. doi: 10.1038/s41598-020-66381-3 (PMC7311386; doi:10.1038/s41598-020-66381-3)
Supplement: Supplementary file 2 — Questionnaire [file 41598_2020_66381_MOESM2_ESM.pdf]

Questionnaire on ASF in domestic pig herds in Romania

The purpose of this survey is to collect epidemiological data as well as any available information on the development of ASF in Romania to:

1. Perform an analysis of the temporal and spatial patterns of ASF in domestic pigs, and
2. Analyze the risk factors involved in the occurrence, spread and persistence of the ASF virus in the domestic population.

INSTRUCTIONS

Please, carefully **read the instructions** before completing the questionnaire.

- A list of control holdings will be provided to the County Directorates by NSVFSA. They were randomly selected from Part III (Decision EC/2014/709) as soon as possible after the confirmation of the matching case holding (outbreak). The control holdings are selected from different affected-Part III counties in Romania and from the same holding type.
- The survey consist on TWO PARTS: one that needs to be filled in BEFORE the visit to the holding BY THE VETERINARIAN appointed to perform the survey, and a second to be filled DURING the visit to the holding.
- In case a holding is selected to perform the survey, but preventive culling has been applied, or the control holding is suspected or has become (in the meanwhile) positive, please **go to the next control holdings on the list** provided by NSVFSA.
- **High Risk Period** is defined as the length of time between introduction and detection of a disease, during which no control measures have yet been implemented and disease continues to spread. In order to harmonize answers, we have arbitrarily defined a time lapse of 2,4 and 6 weeks for each type of holding according to the type of holding. Whenever considering a **High Risk Period question** , consider the number of corresponding weeks.
- Note that you will be asked to provide the GPS location of the holding. Please, be equipped with a device that can retrieve this information (e.g. smartphone), or complete this blank space before the visit.

FIRST PART: TO BE FILLED IN **BEFORE THE VISIT** of the HOLDING BY THE VETERINARIAN appointed to perform the survey

Name of Veterinary Officer appointed to carry out the questionnaire:

Information about the holding:

Holding ID

[ ]

Type of holding to be visited:

- ◯ Non-professional holding
- ◯ Type "A" commercial holding
- ◯ Commercial holding

Disease status of pig herd:

- ◯ Outbreak
- ◯ Control

If outbreak holding, kindly provide the date of ASF confirmation:

[ ]

If control holding, kindly provide the case farm holding ID to which this control farm was associated:

[ ]

County code:

- ◯ AB
- ◯ AR
- ◯ AG
- ◯ BC
- ◯ BH
- ◯ BN
- ◯ BT
- ◯ BV
- ◯ BR
- ◯ B
- ◯ BZ
- ◯ CS
- ◯ CL
- ◯ CJ
- ◯ CT
- ◯ CV
- ◯ DB
- ◯ DJ
- ◯ GL
- ◯ GR
- ◯ GJ
- ◯ HR
- ◯ HD

- IL
- IS
- IF
- MM
- MH
- MS
- NT
- OT
- PH
- SM
- SJ
- SB
- SV
- TR
- TM
- TL
- VS
- VL
- VN

SECOND PART: to be filled in DURING the visit

GPS coordinates

Help yourself with your smartphone location if needed: keep pressed your location on Google maps and a red pin will show up with the coordinates on the upper left search bar.

Date of the visit

Holding animals information

Kindly provide the total number of pigs in the holding:

Please, specify the number of each kind of pigs in the holding:

Piglets:

Breeding sows:

Breeding boars:

Fattening male pigs:

Fattening female pigs:

Where are the pigs slaughtered?

- On the holding
- In your own slaughterhouse
- In a contracted slaughterhouse

Do pigs have access to outdoor areas on your holding?

- Yes
- No

If yes, did pigs have outdoor access during the High Risk Period?

Duration of the High Risk Period according to the farm type:
Non-professional holdings: 2 weeks
"Type A" holdings: 4 weeks
Commercial holding: 6 weeks

- Yes
- No

FOR NON-PROFESSIONAL HOLDINGS: are there other animal species with access to the holding or which are kept in the same pig shed?

- Bovine
- Ovine
- Caprine
- Poultry
- Horses
- Pets (dogs, cats)
- Rabbits
- Other
- No other species

If other, please, specify which

Wild boar information

Have you ever seen a wild boar roaring or moaning near your farm?

- Yes
- No

Do you ever observe crossbred pigs near your holding?

- Yes
- No

Have you ever found / sent a wild boar body / remains in the vicinity of your farm?

- Yes
- No

Could a wild boar access the **feed storage**?

- Yes
- No

Could a wild boar access the **bedding storage**?

- Yes
- No

Are there any attractive crops/feed resources (maize, fruit trees, oak trees, …) around/near the holding?

- Yes
- No

Feed and drinking water

What type of feed is given on your holding?

More than one answer is possible

- Industrial compound feed (1)
- Industrial and/or agricultural by-products
- Forage (e.g. hay, fresh grass, silage) (2)
- Cereals (grains) (3)
- Kitchen waste
- On farm milling and mixture (4)
- Other

(1) If you have checked "COMMERCIAL COMBINED FOODS", please specify the origin of the compound feed (supplier)

(2) Please specify the origin of the forrage

- Cultivated in an ASF-affected area
- Cultivated in an ASF-free area
- I don't know

(3) Please specify the origin of the cereals

- Cultivated in an ASF-affected area
- Cultivated in an ASF-free area

- [ ] I don't know

(4) Please specify the origin of the on-farm milling and mixture

- [ ] Cultivated in an ASF-affected area
- [ ] Cultivated in an ASF-free area
- [ ] I don't know

What kind of drinking water is provided to the animals?

- [ ] Fountain water (pumped from groundwater)
- [ ] Water stored in holding's own reservoir (e.g. tank, container, basin...)
- [ ] River/lake water
- [ ] Tap water (drinking / cleaning / disinfected water)

If you have ticked OTHERS, please specify which:

Biosecurity

The total number of visits made by vehicles in operation during the high risk period:

Duration of the High Risk Period according to the holding type:
Non-professional holdings: 2 weeks
"Type A" holdings: 4 weeks
Commercial holding: 6 weeks

How many **professionals** (veterinarians, consultants, maintenance, ...) entered the holding in total during the High Risk Period?

Duration of the High Risk Period according to the holding type:
Non-professional holdings: 2 weeks
"Type A" holdings: 4 weeks
Commercial holding: 6 weeks

How many **visitors** (family, friends, students, ...) entered the holding in total during the High Risk Period?

Duration of the High Risk Period according to the holding type:
Non-professional holdings: 2 weeks
"Type A" holdings: 4 weeks
Commercial holding: 6 weeks

What kind of bedding is used in the sheds?

- ( ) Straw
- ( ) Wood chips
- ( ) Sawdust
- ( ) None
- ( ) Other

If other, please specify which

Is the holding fenced to avoid contact with wild animals (e.g. wild boar, wolves, foxes, jackals,...)?

- Yes
- No

Is manure from other holdings spread on neighboring farmlands situated directly next to your stables (< 100 meters)?

- Yes
- No

Were NEW pigs and/or piglets introduced in the establishment during the High Risk Period?

Duration of the High Risk Period according to the holding type:
Non-professional holdings: 2 weeks
"Type A" holdings: 4 weeks
Commercial holding: 6 weeks

- Yes
- No

Were there any possible contacts with case farms in the High Risk Period? (e.g. natural mount, artificial insemination, shared material/objects, external visitors, etc.)

Duration of the High Risk Period according to the holding type:
Non-professional holdings: 2 weeks
"Type A" holdings: 4 weeks
Commercial holding: 6 weeks

- Yes
- No
- I am not sure/I do not know

If yes, please specify the type of contact (e.g. vehicles, people, equipment,...):

Does anyone among the workers of your holding carry out any outdoor activity (in wild boar habitat)?

- Work (forest management, agricultural activities, bee-keeping, ...)
- Leisure (hiking, mountain climbing, mountain biking, bird watching,...)
- Hunting
- None of above
- Other

If other, please specify which:

FOR NON-PROFESSIONAL HOLDINGS ONLY: Are boars from other farms used for reproduction on your holding during the High Risk Period?

Duration of the High Risk Period according to the holding type:
Non-professional holdings: 2 weeks

- Yes
- No

Questions **ONLY TO BE ANSWERED FOR Type "A" COMMERCIAL HOLDINGS:**

Can carcasses be collected by the rendering company from the public road, without entering to the premises?

- Yes
- No

Non-contact loading: is it possible for the pigs loaded for slaughter to return to the shed?

- Yes
- No

Are all stables accessible to visitors only through the locker room?

- Yes
- No

Is there a rodent control management plan implemented?

- Yes
- No

Are there intact insect nets placed in front of the air intakes/windows in the sheds?

- Yes
- No

When was an insecticide last applied in your holding? (number of days)

Type 0 if no insecticide is applied;
Write 1 if 0-1 days after applying the last insecticide

What product is used for disinfection of baths/ boot washers/stables at the holding?

Name of commercial product

Do your workers (farm staff, including family) have contact with pigs outside the holding?

- Yes
- No

Is a quarantine period of 30 days respected if new animals arrive or are purchased in your holding?

- Yes

- No

Vectors

Vector ID number

[ ]

Please, can you estimate how many insects of every type did were present at the moment of the visit? (based on visual inspection in the pig shed)

Date on which the measurement was performed

[ ]

Soft ticks (in cracks on the wall or on the ground of pig shed, nearby animals).

- [ ] 0
- [ ] 0-4
- [ ] 5-9
- [ ] 10-100

Hard ticks (on average on an individual pig).

- [ ] 0
- [ ] 0-4
- [ ] 5-9
- [ ] 10-100

Mosquitoes (in the pigs shed, nearby the animals)

- [ ] 0
- [ ] 0-4
- [ ] 5-9
- [ ] 10-100
- [ ] 100-1000
- [ ] >1000

Biting midges (in the pigs shed, nearby the animals)

- [ ] 0
- [ ] 0-4
- [ ] 5-9
- [ ] 10-100
- [ ] 100-1000
- [ ] >1000
